# Influence of Self-Efficacy on Compliance to Workplace Exercise

**DOI:** 10.1007/s12529-012-9239-0

**Published:** 2012-05-24

**Authors:** Mette Merete Pedersen, Mette Kreutzfeldt Zebis, Henning Langberg, Otto Melchior Poulsen, Ole Steen Mortensen, Jette Nygaard Jensen, Gisela Sjøgaard, Thomas Bredahl, Lars Louis Andersen

**Affiliations:** 1 National Research Centre for the Working Environment, Lersø Parkallè 105, 2100 Copenhagen, Denmark; 2 Institute of Sports Medicine Copenhagen, Copenhagen University Hospital, Bispebjerg, 2400 Copenhagen, Denmark; 3 Institute of Sports Science and Clinical Biomechanics, University of Southern Denmark, 5320 Odense, Denmark; 4 Department of Occupational and Environmental Medicine, Copenhagen University Hospital, Bispebjerg, 2400 Copenhagen, Denmark

**Keywords:** Self-efficacy, Physical activity, Compliance

## Abstract

**Background:**

Continuous neck and shoulder pain is a common musculoskeletal complaint. Physical exercise can reduce pain symptoms, but compliance to exercise is a challenge. Exercise-specific self-efficacy has been found to be a predictor of participation in preplanned exercise. Little is known about the influence of exercise-specific self-efficacy on compliance to workplace physical exercise.

**Purpose:**

To determine the influence of exercise-specific self-efficacy on compliance to specific strength exercises during working hours for laboratory technicians.

**Methods:**

We performed a cluster-randomized controlled trial, including laboratory technicians from two industrial production units in Copenhagen, Denmark. The participants were randomized to supervised specific strength exercises for the neck and shoulder muscles for 20 minutes three times a week (*n* = 282) or to a reference group (*n* = 255). The participants answered baseline and follow-up questions regarding self-efficacy and registered all exercises in a diary.

**Results:**

Overall compliance to exercises was 45 %. Compliance in company A (private sector) differed significantly between the three self-efficacy groups after 20 weeks. The odds ratio of compliance was 2.37 for moderate versus low self-efficacy, and 2.93 for high versus low self-efficacy. No significant difference was found in company B (public sector) or in the intervention group as a whole.

**Conclusion:**

We did not find self-efficacy to be a general statistically significant predictor of compliance to exercises during 20 weeks, but found self-efficacy to be a predictor of compliance in a private sector setting. Workplace-specific differences might be present and should be taken into account.

## Introduction

Musculoskeletal pain is one of the most common and costly health problems in Europe and North America [1, 2]. In Denmark, musculoskeletal disorders comprises half of all work-related disorders [3], and continuous neck and shoulder pain is one of the common complaints [1, 2]. Targeted physical exercise for working adults can have positive effects on neck and shoulder pain symptoms [4–7]. However, compliance to exercises is challenging for many employees, and studies comprising interventions during working hours have reported moderate compliance at best [8, 9]. To target health-promoting strategies more efficiently, it is important to identify characteristics associated with low compliance. Self-efficacy has been stated a key predictor of human behavior and describes a person's beliefs in his or her own abilities to make a behavioral change [10–13]. Self-efficacy is defined as: People's judgments of their capabilities to organize and execute courses of action required to attain designated types of performances. It is concerned not with the skills one possesses, but rather with judgments of what one can do with whatever skills one possesses [14].

The self-efficacy theory states that confidence in one's ability to conduct a given task or behavior is strongly related to one's actual ability to perform that behavior [14]. Self-efficacy varies in different domains of functioning and should therefore be used in a domain-specific manner [11, 15]. Exercise self-efficacy has been found to correlate positively with initiation and maintenance of physical exercises especially in the early and middle stages of a preplanned program [16–24] and to be a predictor of general physical activity during leisure time [17, 20, 22, 25]. Additionally, participating in physical activity can improve exercise self-efficacy and subsequently lead to further participation in physical activity [12, 26]. However, little is known about the influence of exercise self-efficacy on compliance to physical exercise in different workplace settings.

Our study determines the influence of exercise-specific self-efficacy on compliance to specific strength exercises for laboratory technicians at two different workplaces from the private and public sector, respectively. We hypothesize that: at both workplaces, (1) individuals with a low initial exercise-specific self-efficacy are less compliant than those with a high initial exercise-specific self-efficacy and that (2) specific strength exercises enhance exercise-specific self-efficacy.

## Material and Methods

### Study Design

We performed a cluster-randomized controlled trial (RCT) in Copenhagen, Denmark (for full methodological description, see Zebis et al. [27]). The present paper includes a prospective observational study on the exercise group within the cluster-RCT, as well as an intention-to-treat analysis on changes in exercise self-efficacy (hereafter, self-efficacy) in the exercise and control groups. We recruited subjects from two industrial production units—a private sector company (A) and a public sector company (B)—in February 2009. Company A was characterized by having strong leadership commitment to social responsibility and health-enhancing activities at work, in particular, regarding physical exercise, and to communicate their strategy to the workers. Many workers performed various leisure time physical activities. Company B, in contrast, did not demonstrate any special leadership involvement in these areas, but they accepted the study to be conducted at the workplace. At both workplaces, the subjects were laboratory technicians performing monotonous and repetitive work. In both companies, the participants were randomized on a cluster level to two different intervention groups: specific strength exercises (SSE) and reference (REF). This cluster randomization resulted in two groups (SSE, *n* = 282; REF, *n* = 255) that were comparable with regard to age, height, and weight. The reference group had a higher proportion of men than the exercise group, which was controlled for in the analysis (for more details, see Zebis et al. [27]). The SSE group consisted of 196 in company A and 86 in company B, and the REF group consisted of 167 in company A and 88 in company B. The analysis of the prospective observational part of the study included the 268 participants of the exercise group who had accepted participation at baseline, and who were not excluded from the study throughout the 20-week study period. The analysis of the cluster-RCT part of the study included participants in the exercise and reference groups who replied to the baseline and follow-up questions regarding self-efficacy. The local ethical committee (HC2008103) approved the study protocol, which was registered in ClinicalTrials.gov (NCT01071980). We informed the participants about the purpose and content of the project, and all participants gave written informed consent to participate in the study, which conformed to the Declaration of Helsinki.

### Intervention

The SSE group performed specific strength exercises locally for the neck and shoulder muscles with five different dumbbell exercises 20 minutes three times a week for 20 weeks during working hours [27]. Experienced instructors introduced the participants to the program. The introduction was held in small groups of 5–15 participants. Hereafter, all participants were allowed to exercise on individual basis or in self-organized groups. Supervision was offered in half of the exercise sessions. The REF group was not offered any physical exercises (results presented elsewhere [27]).

### Compliance

The participants logged all exercises in a diary. Subsequently, compliance was determined as the total amount of exercise sessions during the study period (0–60 sessions) and categorized into the following: (1) high compliance = 40–60 sessions, (2) moderate compliance = 20–39 sessions, (3) low compliance = 10–19 sessions, and (4) very low compliance = 0–9 sessions. This quartering was based on previous definitions of regular, irregular, and seldom participation [28].

### Self-efficacy

The participants replied to a questionnaire on self-efficacy at baseline and at the 20-week follow-up. Self-efficacy was determined with a scale consisting of six items [29–32]. The items were “I'm confident that I'm able to exercise 20 min three or more times per week even if…”: “I am under a lot of stress,” “I feel I don't have the time,” “I have to exercise alone,” “I don't have access to exercise equipment,” “I am spending time with friends or family who do not exercise,” and “It's raining or snowing” [29–32]. Self-efficacy was scored on a five-point Likert scale [33] with response categories from “not at all confident” to “completely confident.” The score of the scale was obtained by summing the scores of the six items [29–32]. In the analysis of the data, the participants were grouped according to their self-efficacy score at baseline into low self-efficacy (6–14 points), moderate self-efficacy (15–22 points), and high self-efficacy (23–30 points). Self-efficacy as a predictor of compliance was tested after 10 and 20 weeks.

### Control Variables

Age, gender, and baseline neck/shoulder pain were used as control variables. To determine pain intensity in the neck and shoulder region, participants replied to the following question: “On a 0–9 scale (where 0 means no complaints, and 9 means pain as bad as it can be), what degree of pain or discomfort have you experienced in (body part) during the last three months?” The question was asked with body part replaced first by the neck, then by the left shoulder, and then by the right shoulder. The variable “baseline neck/shoulder pain” was defined as the maximum of the three reported pain intensities.

### Statistical Analysis

In the prospective observational study on the exercise group within the cluster-RCT, we used cumulative logistic regression to model the odds for compliance as a function of self-efficacy. The analysis was controlled for gender, age (<40, 40–49, and ≥50 years), and baseline pain (0, 1–2, and ≥3). Intracluster correlations were handled by treating individual observations within a cluster as repeated measurements in a generalized estimating equations model. The calculations were performed, in SAS version 9.1, by use of the GENMOD procedure. Parameter estimates and standard errors were based on the empirical covariance matrix. We first treated the self-efficacy groups as qualitative variables and estimated odds ratios for compliance. People in the category “low self-efficacy” were used as reference. We, thereafter, tested the hypothesis that compliance increases with self-efficacy. In the hypothesis test, we treated self-efficacy as a numeric variable with the value 1 for people in the category “low,” 2 for the category “moderate,” and 3 for the category “high self-efficacy.” As the hypotheses were one-sided, the significance level was set to 0.10.

In the intention-to-treat design, a two-way analysis of variance (group; time) was performed to test for the effect of exercises versus reference on changes in self-efficacy at 20 weeks. To determine the robustness of self-efficacy over time, Spearman's correlation coefficient between baseline and follow-up self-efficacy was calculated. The hypothesis test was two-sided with a significance level of 0.05.

## Results

### Participation

On average, the participants performed 27 out of the 60 exercise sessions during the 20 weeks and thus participated in 45 % of the exercise sessions, and 22 % (*n* = 58) of the participants exercised less than 10 times; 14 % (*n* = 37) exercised 10–19 times; 31 % (*n* = 84) exercised 20–39 times, and 33 % (*n* = 89) exercised 40 times or more.

### Pooled Analysis for the Two Companies

Participants in the low self-efficacy group (*n* = 35) exercised on average 25.6 times: The corresponding numbers for the categories moderate and high where 27.4 (*n* = 115) and 28.0 (*N* = 117). In the intervention group as a whole (pooled for the two participating companies), compliance did not differ significantly between the three self-efficacy groups after 10 weeks nor after 20 weeks. After 10 weeks, the cumulative odds ratio of compliance was 1.40 for moderate versus low self-efficacy and 1.34 for high versus low self-efficacy. The trend was nonsignificant (*P* = 0.24). Compliance did not differ significantly between the three self-efficacy groups after 20 weeks (*P* (positive trend) = 0.24). The odds ratio of compliance was 1.31 (95 % confidence interval (CI), 0.66–2.60) for moderate versus low self-efficacy and 1.43 (95 % CI, 0.63–3.26) for high versus low self-efficacy.

### Company Level Analysis

**Fig. 1:**
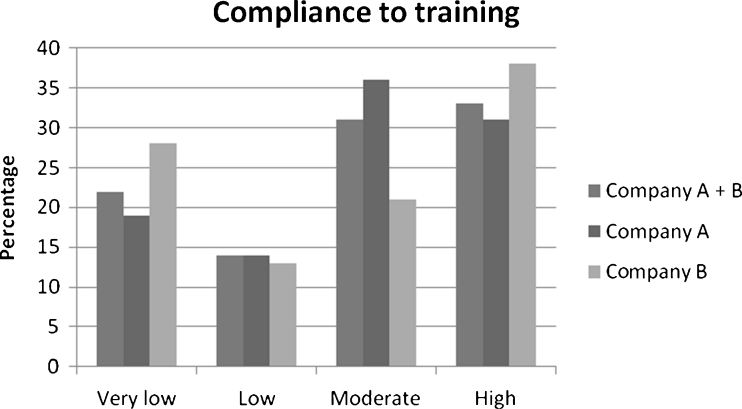
Compliance to training in the intervention group as a whole and in companies A and B separately

**Fig. 2:**
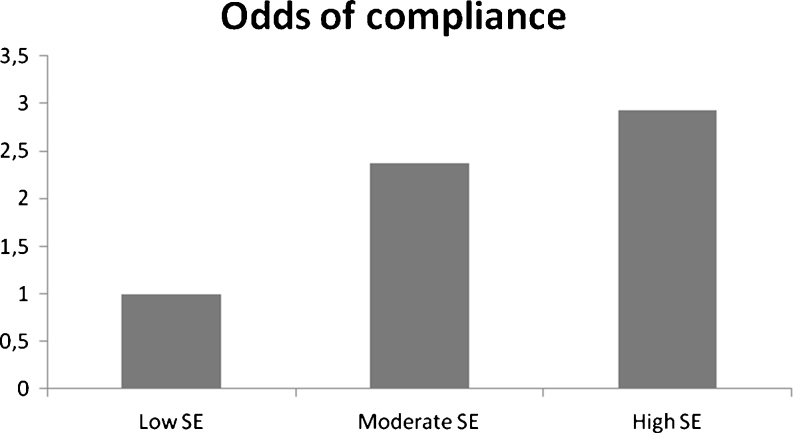
Odds of compliance for the three self-efficacy groups in company A. Low self-efficacy was set as reference group (odds = 1)

We also analyzed data for each participating company separately. The participants in company A exercised on average 28 times during the 20 weeks, and 19 % (*n* = 35) exercised less than 10 times; 14 % (*n* = 27) exercised 10–19 times; 36 % (*n* = 68) exercised 20–39 times, and 31 % (*n* = 59) exercised 40 times or more (Fig. 1). Compliance in company A differed significantly between the three self-efficacy groups after 20 weeks. After 10 weeks, the cumulative odds ratio of compliance was 2.23 for moderate versus low self-efficacy and 2.55 for high versus low self-efficacy. The trend was not statistically significant (*P* (trend) = 0.06). The difference between the three self-efficacy groups after 20 weeks was statistically significant (*P* (positive trend) = 0.04). The odds ratio of compliance was 2.37 (95 % CI, 1.13–4.97) for moderate versus low self-efficacy and 2.93 (95 % CI, 1.66–7.98) for high versus low self-efficacy (Fig. 2).

The participants in company B exercised on average 26 times during the 20 weeks, and 28 % (*n* = 22) exercised less than 10 times; 13 % (*n* = 10) exercised 10–19 times; 21 % (*n* = 17) exercised 20–39 times, and 38 % (*n* = 30) exercised 40 times or more (Fig. 1). In company B, compliance to exercises as a function of self-efficacy at baseline was neither significant at week 10 nor at week 20. Based on these results, we made an exploratory analysis for a possible interaction between company and self-efficacy, which turned out to be significant (*P* = 0.02). This test validated our observation of self-efficacy being significant at one, but not the other company.

### Intention-to-Treat Analysis

We also tested for the effect of exercises versus reference on changes in self-efficacy (scale 6–30 points) at 20 weeks, but no significant effects were observed, neither at company A (between-group difference, 0.1; 95 % confidence interval, −1.2 to 1.4), company B (between-group difference, −0.2; 95 % confidence interval, −1.5 to 1.2), nor at both companies analyzed together (between-group difference, 0.4; 95 % CI, −3.1 to 4.0). A spearman correlation analysis between self-efficacy at baseline and follow-up showed a correlation coefficient of 0.56 (control group, *P* < 0.0001) and 0.55 (exercise group, *P* < 0.0001).

## Discussion

We did not find self-efficacy to be a general predictor of compliance to specific strength exercises at the workplace. Self-efficacy may be a predictor of compliance to specific strength exercises in some workplaces, but not in others. The present study included a private and a public sector company, and when analyzed separately, self-efficacy was found to be a predictor of compliance during 20 weeks in the private sector company, but not in the public sector company. There was no significant effect of exercises versus reference on changes in self-efficacy over 20 weeks, and self-efficacy did not change over time in any group.

Self-efficacy has been shown to predict physical activity in a series of observational studies where the research did not influence the physical activity level of the study participants [17, 18, 20, 25] and has also been found to be a predictor in experimental settings where the outcome was compliance to one or more predefined exercise programs [21, 22]. This study did not find baseline self-efficacy to be a predictor of compliance to training over time in the intervention as a whole. However, we did find self-efficacy to be a predictor of compliance in the private sector company, but not in the public sector company. Workplace-specific differences that need to be taken into account, when considering compliance, might therefore be present. Barriers towards exercising are multidimensional and can be both population and workplace specific [34]. These barriers might be lack of time, lack of support from company leaders and colleagues, etc. Studies have identified barriers towards exercising at the workplace like work load, limited break time, time wise scheduling, and work conflicts [35, 38]—factors that might have influenced compliance differently at the two companies in the present study. The limited predictive value of self-efficacy may also indicate that other individual factors and factors other than specific individual psychological factors like self-efficacy may be more important in predicting exercise behavior. The social cognitive theory specifies a set of core determinants for health behavior. These include knowledge of health risks and benefits, perceived self-efficacy, expectancies about the consequences of one's actions, personal goals, and perceived impediments [15]. Thus, personal psychological factors other than self-efficacy may play a role regarding exercise participation. A specific worksite atmosphere as well as social and cultural factors, like social class, peers, and environment, may better explain barriers or motivation for exercise than initial self-efficacy. Moreover, interactions between participants and social support from group members influence a person's health behavior [11, 13, 15]. The personality and commitment of the instructor and the contact person of the exercise group may also have influenced compliance. In future research, a more systemic approach could encompass these factors and possibly provide knowledge of important issues to ensure compliance to interventions and, furthermore, to better understand barriers and motivation for exercise and physical activity. In this study, participants with low self-efficacy had a lower compliance than participants with moderate and high self-efficacy. Compliance did not differ between the latter two groups. Thus, self-efficacy to a small extend is not sufficient in securing compliance, which should be taken into account when planning exercise regimens.

The prospective design and an analysis controlling for factors that might be related to compliance strengthen our study. Apart from age and gender, we controlled for baseline neck and shoulder pain considering that the aim of the study was to reduce neck and shoulder pain. In contrast to previous studies, we also took intracluster correlations into account by treating individual observations within the exercise groups as repeated measurements. A weakness of research studies focusing on exercise is the dependence on volunteers. People with extremely low self-efficacy towards exercising would probably not volunteer for an exercise program. Employees who accept participation might therefore have a higher degree of exercise-specific self-efficacy than those who do not volunteer, creating a risk of a selected group with a smaller difference in self-efficacy than in the population as a whole. The low self-efficacy group in the present study (*n* = 35) was smaller than the moderate (*n* = 115) and the high self-efficacy (*n* = 118) groups. Compliance as well as self-efficacy among the participants might have been influenced by the presence of instructors and by the workplace design of the study, which limits the external validity to workplace settings involving training instructors. Thus, the results cannot be generalized to exercise behavior where no instructors are present nor to the population as a whole. A limitation of the study is the use of a self-efficacy measure on generic exercise self-efficacy even though adapted in wording to the workplace context. A workplace-specific measure might have shown another picture of self-efficacy as a predictor of compliance. The measure, though, does reflect an individual's efficacy belief regarding initiation and maintenance of exercise. Even though self-efficacy is an action-specific measure, it might also well reflect a fundamental view on exercise participation and thus on exercising at the workplace.

## Conclusion

In conclusion, we did not find self-efficacy to be a general predictor of compliance to workplace exercise. However, we did find self-efficacy to be a predictor of compliance in a private sector setting. The limited predictive value of self-efficacy may indicate that other than specific individual psychological factors like self-efficacy may be important in predicting exercise behavior. Further research in this area is needed to understand what predicts compliance to workplace exercise programs and to clarify barriers and motivation for training at different workplaces in order to target future preventive strategies more efficiently. A systemic approach, including psychological, cultural, and social factors, could provide useful knowledge in clarifying the aspects of compliance.
